# Lower Viral Loads and Slower CD4^+^ T-Cell Count Decline in MRKAd5 HIV-1 Vaccinees Expressing Disease-Susceptible HLA-B*58:02

**DOI:** 10.1093/infdis/jiw093

**Published:** 2016-03-06

**Authors:** Ellen M. Leitman, Jacob Hurst, Masahiko Mori, James Kublin, Thumbi Ndung'u, Bruce D. Walker, Jonathan Carlson, Glenda E. Gray, Philippa C. Matthews, Nicole Frahm, Philip J.R. Goulder

**Affiliations:** 1 Department of Paediatrics; 2 Nuffield Department of Medicine, University of Oxford, United Kingdom; 3 HIV-1 Vaccine Trials Network, Vaccine and Infectious Disease Division, Fred Hutchinson Cancer Research Center; 4 Department of Global Health, University of Washington, Seattle; 5 eScience Group, Microsoft Research,Redmond, Washington; 6 Ragon Institute of MGH, MIT, and Harvard, Cambridge, Massachusetts; 7 HIV-1 Pathogenesis Programme, Doris Duke Medical Research Institute, University of KwaZulu-Natal; 8 KwaZulu-Natal Research Institute for Tuberculosis and HIV-1, University of KwaZulu-Natal, Durban; 9 South African Medical Research Council, Cape Town; 10 Perinatal HIV-1 Research Unit, University of the Witwatersrand, Johannesburg, South Africa; 11 Max Planck Institute for Infection Biology, Berlin, Germany

**Keywords:** HLA class I, HIV-1 vaccine, Phambili trial, Gag-specific CD8+ T cells

## Abstract

***Background.*** HLA strongly influences human immunodeficiency virus type 1 (HIV-1) disease progression. A major contributory mechanism is via the particular HLA-presented HIV-1 epitopes that are recognized by CD8^+^ T-cells. Different populations vary considerably in the HLA alleles expressed. We investigated the HLA-specific impact of the MRKAd5 HIV-1 Gag/Pol/Nef vaccine in a subset of the infected Phambili cohort in whom the disease-susceptible HLA-B*58:02 is highly prevalent.

***Methods.*** Viral loads, CD4^+^ T-cell counts, and enzyme-linked immunospot assay–determined anti-HIV-1 CD8^+^ T-cell responses for a subset of infected antiretroviral-naive Phambili participants, selected according to sample availability, were analyzed.

***Results.*** Among those expressing disease-susceptible HLA-B*58:02, vaccinees had a lower chronic viral set point than placebo recipients (median, 7240 vs 122 500 copies/mL; *P* = .01), a 0.76 log_10_ lower longitudinal viremia level (*P* = .01), and slower progression to a CD4^+^ T-cell count of <350 cells/mm^3^ (*P* = .02). These differences were accompanied by a higher Gag-specific breadth (4.5 vs 1 responses; *P* = .04) and magnitude (2300 vs 70 spot-forming cells/10^6^ peripheral blood mononuclear cells; *P* = .06) in vaccinees versus placebo recipients.

***Conclusions.*** In addition to the known enhancement of HIV-1 acquisition resulting from the MRKAd5 HIV-1 vaccine, these findings in a nonrandomized subset of enrollees show an HLA-specific vaccine effect on the time to CD4^+^ T-cell count decline and viremia level after infection and the potential for vaccines to differentially alter disease outcome according to population HLA composition.

**Clinical Trials Registration.** NCT00413725, DOH-27-0207-1539.

HLA class I alleles are an important predictor of disease course in human immunodeficiency virus type 1 (HIV-1) infection [1]. In sub-Saharan Africa, HLA-B*57, HLA-B*58:01, and HLA-B*81:01 are protective against disease progression, while HLA-B*18:01 and HLA-B*58:02 are associated with rapid progression [2–4]. One contributory factor is the role of HLA molecules in presenting particular epitopes to induce CD8^+^ T-cell responses against HIV-1. Gag-specific CD8^+^ T-cell responses are associated with lower viremia levels and are dominant in people with protective HLA alleles, such as HLA-B*57:03 [1, 5]. In contrast, disease-susceptible HLA alleles, such as HLA-B*58:02, typically present relatively ineffective non-Gag responses [6] associated with high viral set points [5]. This prompts the hypothesis that a successful vaccine might induce Gag-specific responses in subjects who would not target this protein in natural infection.

Two recent randomized efficacy trials of the MRKAd5 subtype B HIV-1 Gag/Pol/Nef T-cell vaccine provide a unique opportunity to address this hypothesis [7, 8]. The first study, Step, was conducted in the Americas, Australia, and the Caribbean, where HIV-1 subtype B predominates [7]. Both interim and follow-up analyses reported vaccine-enhanced HIV-1 acquisition [7, 9]. The second trial, Phambili, tested the efficacy of the same vaccine in South Africa, where HIV-1 subtype C is endemic [8, 10]. Following Step discontinuation, Phambili was terminated 9 months into the study, having enrolled 801 subjects (27%) of the originally scheduled 3000 [10]. As in Step, the vaccine increased the risk of HIV-1 acquisition and did not reduce viremia levels in early infection [8, 10].

Post hoc Step studies, however, demonstrated lower viremia levels in vaccinees with a greater breadth of Gag-specific CD8^+^ T-cell activity [11], consistent with the notion of Gag-specific efficacy, but also suggested the possibility of lower viral loads in vaccinees expressing protective HLA alleles [12, 13]. To examine this question in a population substantially dissimilar in the HLA alleles expressed from that studied in Step, we here tested in the South African Phambili cohort the hypothesis that the MRKAd5 HIV-1 vaccine might indeed have an HLA-specific effect.

Specifically, we hypothesized that subjects expressing HLA-B*58:02, the most prevalent HLA-B allele in South Africans [2, 5], might benefit from the MRKAd5 HIV-1 vaccine. The rationale was that the HLA-B*58:02–restricted CD8^+^ T-cell response is solely Env specific and, therefore, associated with poor immune control [5, 14]; HLA-B*58:02 indeed is strongly associated with rapid HIV-1 disease progression in southern Africa [2, 3, 6]. Hence, vaccine-mediated induction of Gag-specific responses in subjects expressing HLA-B*58:02 would, hypothetically, improve disease course in vaccinees who subsequently became HIV-1 infected. HLA-B*18:01, also strongly associated with rapid progression [2, 3], restricts a dominant CD8^+^ T-cell epitope in Nef [5]. Although Nef-specific CD8^+^ T-cell responses have also been associated with poor control of HIV-1 [5, 15–17], the immunodominance of the HLA-B*18:01–restricted CD8^+^ T-cell response might be unaffected by the MRKAd5 HIV-1 Gag/Pol/Nef vaccine.

We set out, therefore, to investigate the impact of the MRKAd5 HIV-1 vaccine in the Phambili trial, particularly on the HLA-B*58:02–expressing individuals, to determine whether immune control of HIV-1 was improved in vaccinees who subsequently became infected. This study has limitations that we wish to highlight, including postrandomization bias, sample selection bias, and low subject numbers (see Discussion), but it provides potentially important insights into HLA-dependent vaccine effects on the postinfection disease course and immune response to HIV-1.

## METHODS

### Study Design

The Phambili Ancillary Study included 100 HIV-1–infected Phambili participants (Supplementary Figure 1). Sixty of them, for whom HLA types, peripheral blood mononuclear cell (PBMC), viral load, and CD4^+^ T-cell count data were available, compose the focus of this report (Table 1). For the remaining 40 individuals, no PBMC were available to assess CD8^+^ T-cell responses, and thus only selected viral load and CD4^+^ T-cell count data were included in additional analyses; material for HLA typing was available for 25 participants. Subjects were categorized as having protective HLA-B expression (HLA-B*57:02/57:03/58:01/81:01); disease-susceptible HLA-B expression (HLA-B*18:01/58:02), and neither protective nor disease-susceptible HLA-B expression (eg, HLA-B*42:01/44:03) [2, 3]. For the most prevalent HLA-B allele, HLA-B*58:02 (phenotypic frequency, 23%), there were sufficient subject numbers to compare vaccinees (n = 7) to placebo recipients (n = 7). All participants provided written informed consent; ethics committee at each clinical site and the University of Oxford approved the study.

**Table 1.** Baseline Characteristics of the 60 Infected Phambili Participants Studied in This Report

Characteristic	Vaccine	Placebo	Total
Subjects	37 (62)	23 (38)	60 (100)
Age at enrollment, y	24 (21.5–28.5)	24 (22–32)	24 (22–29)
Sex			
Female	21 (57)	18 (78)	39 (65)
Male	16 (43)	5 (22)	21 (35)
Study site			
Soweto, Johannesburg	15 (41)	7 (30)	22 (37)
Emavundleni, Cape Town	8 (22)	6 (26)	14 (23)
CAPRISA, Klerksdorp	5 (14)	4 (17)	9 (15)
eThekwini, Durban	7 (19)	3 (13)	10 (17)
Medunsa, Polokwane	2 (5)	3 (13)	5 (8)
Time since diagnosis, mo.			
Time point 1	2 (1–2.5)	2 (1–3)	2 (1–3)
Time point 2	12 (11–12)	11 (11–12)	12 (11–12)
Duration of follow-up data, time since diagnosis, mo.	17 (16–18)	17 (16–19)	17 (16–18)
Started ART	5 (14)	6 (26)	11 (18)
HLA type^a^			
Protective			
B*57:03	12 (32)	3 (13)	15 (25)
B*58:01	2 (5)	0 (0)	2 (3)
B*81:01	7 (19)	3 (13)	10 (17)
Disease susceptible	4 (11)	0 (0)	4 (7)
B*18:01	0 (0)	1 (4)	1 (2)
B*58:02	7 (19)	7 (30)	14 (23)
Neither protective nor disease-susceptible	18 (49)	13 (57)	31 (52)
Early viral set point,^b^ copies/mL	41 850 (3 675–234 075)	88 500 (11 485–238 500)	71 700 (6 475–238 500)

Data are no. (%) of subjects or median value (interquartile range). There were no significant differences between vaccine and placebo groups for any of the characteristics.

Abbreviation: ART, antiretroviral therapy.

^a^ HLA types were grouped by their association with disease progression, as specified in “Methods” section.

^b^ Defined as the median of human immunodeficiency virus type 1 load measurements 2–3 months after diagnosis, as specified in “Methods” section.

To identify associations between HLA expression and recognition of HIV-1–specific peptides, we used enzyme-linked immunospot (ELISPOT) data from 1031 untreated subjects with chronic natural HIV-1 infection from the previously described Sinikithemba (enrolled in 2003–2008) and Gateway (enrolled in 2012–2013) cohorts in Durban, South Africa [5, 18]. The median viral load in these subjects was 25 700 copies/mL (range, 3690–119 000 copies/mL), and the median CD4^+^ T-cell count was 404 cells/mm^3^ (range, 273–549 cells/mm^3^). PBMCs from each individual were screened with a panel of peptides spanning the entire C-clade 2001 consensus sequence [5] and analyzed (Supplementary Materials). To compare the CD8^+^ T-cell responses in the HLA-B*58:02–positive South African Phambili subjects to those in HLA-B*58:02–positive South African subjects naturally infected with HIV-1, we used ELISPOT data from 66 HLA-B*58:02–positive subjects from the Sinikithemba cohort because individuals in both groups would have had a similar geographical location, genetic background, timing of infection, and infection with similar/related viral species (Supplementary Table 3).

### Interferon γ (IFN-γ) ELISPOT Assays

Blinded to the vaccine/placebo treatment assignments, we screened PBMCs from the 60 subjects with available cells to quantify IFN-γ ELISPOT responses to pools (n = 36) of overlapping peptides spanning the entire C-clade HIV-1 proteome, based on the 2001 C-clade consensus [5]. For each subject, cells obtained 2 and 12 months after diagnosis (Table 1) were tested [19, 20]. Responses were divided into 4 groups: specific to Gag, specific to Pol, specific to Nef, or specific to other (Env, Tat, Rev, Vpu, Vpr, and Vif) proteins. Breadth was defined as the number of pools testing positive (>50 spot-forming cells [SFCs]/10^6^ PBMCs, after background subtraction).

### Statistical Analysis

Baseline characteristics (Table 1) between vaccine and placebo recipients were analyzed using the Mann–Whitney *U* test or the Fisher exact test (Prism v5.0c). Only pre-ART viral loads were used. Early viral set point was defined as the geometric mean of HIV-1 load measurements 2–3 months after diagnosis [7, 10]. To determine the viral set point more accurately, we also defined the long-term chronic set point as the median of all pre-ART viral loads available as (1) including the diagnostic (acute) measurement, (2) excluding the diagnostic measurement, (3) including all measurements from >2 weeks after diagnosis, (4) including all measurements from >1 month after diagnosis, or (5) including all measurements from >3 months after diagnosis. Chronic set points were very similar irrespective of the definition, and results using definition 4 are reported here because this may most accurately reflect the beginning of the chronic phase [21, 22]. Set-point differences between vaccinees and placebo recipients were analyzed using the Mann–Whitney *U* test (Prism v5.0c).

Linear mixed-effects models were constructed to investigate the effects of vaccination, sex, age, anti–adenovirus serotype 5 (Ad5) antibody levels, and herpes simplex virus type 2 (HSV-2) serostatus on longitudinal viremia levels. Random intercept models were constructed with the grouping by participant identifier in R, using the lme4 library [23]; *P* values were obtained by comparing models with and those without vaccination as a fixed effect (analysis of variance [ANOVA]) [24].

The log-rank test (Prism v5.0c) and univariate Cox regression models (SPSS v22.0) were used to compare time to disease progression between vaccine and placebo groups, defined by a CD4^+^ T-cell count of <350 cells/mm^3^, the World Health Organization's CD4^+^ T-cell count criterion for ART initiation [25]. Only pre-ART CD4^+^ T-cell counts were included; the impact of sex, age, Ad5 antibody levels, and HSV-2 serostatus was checked.

Differences in ELISPOT responses between vaccinees and placebo recipients were analyzed in Prism v5.0c by the Mann–Whitney *U* test (2-group analyses) or the Kruskal–Wallis test (3-group analyses).

To identify associations between expression of HLA alleles and recognition of HIV-1–specific peptides, we used a decision tree based on the Fisher exact test (Supplementary Materials) [26].

## RESULTS

### HLA-Independent Vaccine Effects

**Figure 1.**
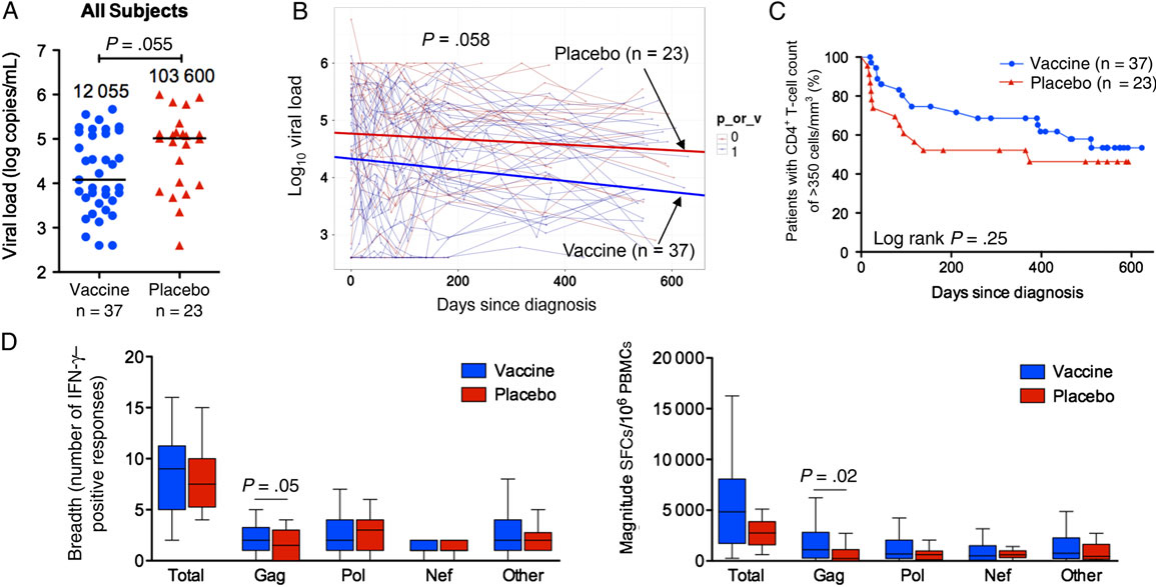
Longitudinal viremia levels, CD4^+^ T-cell counts, and CD8^+^ T-cell responses in the 60 infected vaccinees and placebo recipients, irrespective of HLA. *A*, Chronic viral set point (defined for each individual as the median of all measurements from >3 months after diagnosis). Each patient's set point is shown as a circle (vaccine recipients) or triangle (placebo recipients), and horizontal lines and numbers denote median values. Statistical analysis was performed by the Mann–Whitney *U* test. *B*, Examination of the effect of vaccination on longitudinal viremia levels before antiretroviral therapy initiation. The slopes (thick lines) generated from a linear mixed-effects model are plotted on top of data points and viral load trajectories for individual patients (thin lines). Statistical analysis was performed by analysis of variance. *C*, Kaplan–Meier curves showing time to reach a CD4^+^ T-cell count of <350 cells/mm^3^ in vaccinees and placebo recipients. Statistical analysis was performed by the log-rank test. *D*, Breadth and magnitude of CD8^+^ T-cell responses at the second time point in vaccinees and placebo recipients. In Tukey box plots, horizontal lines indicate median values, boxes show interquartile ranges, and whiskers show minimum and maximum values. Statistical analysis was performed by the Mann–Whitney *U* test. The column termed “Total” shows the combined values of responses to all proteins; the column termed “Other” includes responses to Env, Rev, Tat, Vpu, Vif, and Vpr. Abbreviations: IFN-γ, interferon γ; PBMC, peripheral blood mononuclear cell; SFC, spot-forming cells.

We first compared the baseline characteristics between vaccine and placebo recipients in the selected subgroup of 60 subjects for whom PBMCs were available to test the impact of the MRKAd5 HIV-1 vaccine on postinfection CD8^+^ T-cell activity. These showed no significant differences in age, sex, HLA-B genotype, or early viral set point (Table 1). As previously reported [7, 10, 27], median early viral set point did not differ significantly between vaccine and placebo recipients among these 60 patients (41 850 copies/mL [interquartile range {IQR}, 3657–234 075 copies/mL] vs 88 500 copies/mL [IQR, 11 485–238 500 copies/mL], *P* = .34; Table 1). We observed a trend toward a lower median chronic viral set point in vaccinees in the 60 subjects (12 055 copies/mL [IQR, 3805–137 500 copies/mL] vs 103 600 copies/mL [IQR, 9190–141 800 copies/mL], *P* = .055; Figure 1*A*). However, this difference did not reach statistical significance, even when all of the viral load data were analyzed using a linear mixed-effects model (*P* = .058; Figure 1*B*). It is important to take note of the fact that 12 vaccinees expressed protective alleles (HLA-B*57/58:01/81:01), compared with 3 placebo recipients, although this also did not reach statistical significance (*P* = .13). Additionally, there was no significant difference in time to disease progression (ie, time to a CD4^+^ T-cell count of <350cells/mm^3^) between vaccine and placebo groups (*P* = .25; Figure 1*C*).

Examining the impact of the MRKAd5 HIV-1 vaccine on CD8^+^ T-cell IFN-γ responses after infection in Phambili subjects with available PBMCs, we observed that vaccinees had significantly higher Gag-specific breadth and magnitude than placebo recipients (*P* = .03 and *P* = .02, respectively, 2 months after diagnosis; and *P* = .05 and *P* = .02, respectively, 12 months after diagnosis; Figure 1*D*). These assays were undertaken blinded to the subjects' vaccination assignments. There was no difference between the 2 groups in the responses toward proteins not included in the vaccine (Env, Vif, Vpr, Vpu, Tat, and Rev). However, there was no significant difference in the Pol- or Nef-specific responses between vaccine and placebo recipients either, despite *pol*/*nef* inclusion in the vaccine.

### HLA-Specific Vaccine Effects on Viral Load and CD4^+^ T-Cell Counts: Protective HLA Alleles

**Figure 2.**
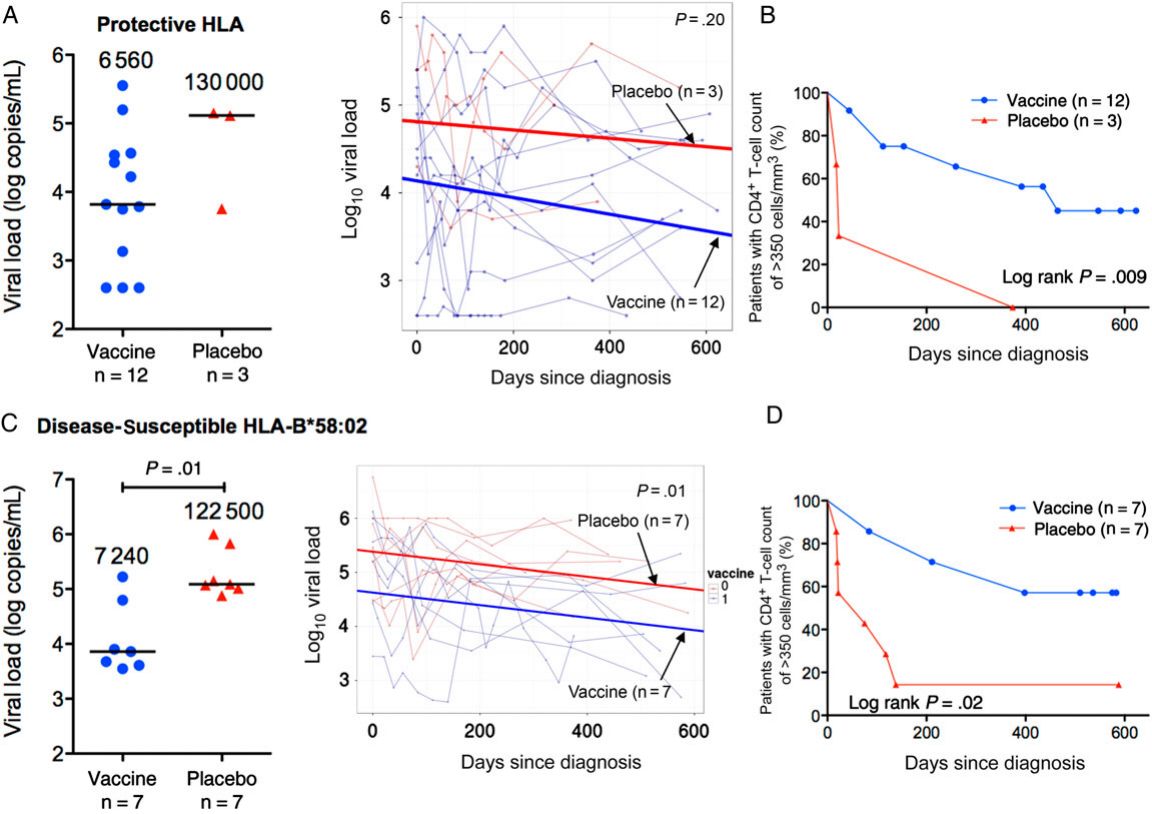
Chronic viral set point and CD4^+^ T-cell counts in infected Phambili subjects expressing protective HLA-B*57/58:01/81:01 or disease-susceptible HLA-B*58:02 alleles. *A–C*, Data for the participants expressing protective alleles (12 vaccinees and 3 placebo recipients). *D–F*, Data for the participants expressing HLA-B*58:02 (7 vaccinees and 7 placebo recipients). *A* and *C*, Chronic viral set point (defined for each individual as the median of all measurements from >3 months after diagnosis) is shown at left. Each patient's set point is shown as circles (vaccine recipients) or triangles (placebo recipients). Horizontal lines and numbers denote median values for vaccine and placebo groups. Statistical analysis was performed by the Mann–Whitney *U* test. Examination of the effect of vaccination on longitudinal viral load values before antiretroviral therapy initiation is shown at right. The slopes (thick lines) generated from a linear mixed-effects model are plotted on top of data points and viral load trajectories (thin lines) for individual patients. Statistical analysis was performed by analysis of variance. *B* and *D*, Kaplan–Meier curves showing time to reach a CD4^+^ T-cell count of <350 cells/mm^3^ in vaccinees and placebo recipients. Statistical analysis was performed by the log-rank test.

To investigate whether these vaccine-mediated effects on the CD8^+^ T-cell responses were associated with HLA-specific immune control, as observed for protective HLA alleles in the Step trial [12], we first compared chronic viral set points and time to a CD4^+^ T-cell count of <350 cells/mm^3^ in the subset of infected Phambili subjects expressing any of the protective alleles HLA-B*57/58:01/81:01 (Table 2). Although subject numbers provided limited power (3 placebo recipients vs 12 vaccinees expressed HLA-B*57/58:01/81:01), there was evidence of vaccine-mediated immune protection through these HLA alleles (Figure 2*A* and 2*B*), with a significantly more-rapid time to disease progression (ie, a CD4^+^ T-cell count of <350 cells/mm^3^) in placebo recipients (*P* = .009). Placebo/vaccine treatment assignment was the only significant contributor to this difference in a univariate analysis that included sex, age, Ad5 antibody levels, and HSV-2 status (Supplementary Table 1).

**Table 2.** Demographic Characteristics Stratified by HLA Group

Characteristic	Protective HLA		Neither Protective nor Disease-Susceptible HLA		Disease-Susceptible HLA-B*58:02	
	Vaccine (n = 12)	Placebo (n = 3)	Vaccine (n = 18)	Placebo (n = 13)	Vaccine (n = 7)	Placebo (n = 7)
Age at enrollment, y	25.5 (22.5–30.5)	32 (22–32)	22.5 (21.5–30.5)	23 (22–26)	22 (21–27)	26 (21–34)
Sex						
Female	7 (58)	3 (100)	9 (50)	10 (77)	5 (71)	5 (71)
Male	5 (42)	0 (0)	9 (50)	3 (23)	2 (29)	2 (29)
Circumcision status^a^						
Circumcised	2 (40)	…	5 (56)	1 (33)	0 (0)	0 (0)
Uncircumcised	3 (60)	…	4 (44)	2 (67)	2 (100)	2 (100)
Adenovirus 5 status^b^						
Seropositive	9 (75)	2 (67)	17 (94)	12 (92)	6 (86)	4 (57)
Seronegative	3 (25)	1 (33)	1 (6)	1 (8)	1 (14)	3 (43)
HSV-2 status at enrollment						
Positive	9 (75)	2 (67)	10 (56)	5 (38)	2 (29)	5 (71)
Negative	3 (25)	1 (33)	8 (44)	8 (62)	5 (71)	2 (29)

Data are no. (%) of subjects or median value (interquartile range). There were no significant differences between vaccine and placebo groups within each HLA subset for any characteristic.

Abbreviation: HSV-2, herpes simplex virus type 2.

^a^ Data are for men circumcised at baseline or during study but before infection and those uncircumcised throughout study or before infection.

^b^ Seropositivity was defined as a titer of >18, and seronegativity was defined as a titer of ≤18.

### HLA-Specific Vaccine Effects on Viral Load and CD4^+^ T-Cell Counts: Disease-Susceptible HLA Alleles

With respect to the disease-susceptible HLA alleles, HLA-B*18:01 and HLA-B*58:02, none of the vaccinees with available PBMCs and HLA data expressed HLA-B*18:01, and analyses were limited to the HLA-B*58:02–positive subjects (7 vaccinees and 7 placebo recipients; Table 2). Median chronic viral set point was significantly lower in HLA-B*58:02–positive vaccinees (7240 copies/mL [IQR, 4090–63 000 copies/mL] vs 122 500 copies/mL [IQR, 103 600–677 500 copies/mL]; *P* = .01; Figure 2*C*). This difference was especially clear when all pre-ART longitudinal data points were interrogated using a mixed-effects model (*P* = .01; Figure 2*C*). These data show that vaccination in the HLA-B*58:02–positive subjects on average resulted in a 0.76 log_10_ reduction in chronic viral set point and that no other factors (sex, age, Ad5 antibody levels, and HSV-2 serostatus) had a significant influence. The viral load differences between vaccinees and placebo recipients remained significant when the 3 additional HLA-B*58:02–positive subjects were added from the Phambili participants with determined HLA types but unavailable PBMCs (*P* = .03, by the Mann–Whitney *U* test; *P* = .03, by an ANOVA mixed-effects model).

Progression to a CD4^+^ T-cell count of <350 cells/mm^3^ was also significantly slower in HLA-B*58:02–positive vaccinees as compared to placebo recipients (*P* = .02, by the log-rank test [Figure 2*D*]; hazard ratio [HR], 0.22 [95% CI, .05–.91; *P* = .04, by a Cox regression model; Supplementary Table 2]). Again, the only significant contributor to this difference was placebo /vaccine treatment assignment, and the difference between vaccine and placebo recipients remained significant with addition of the 3 HLA-B*58:02–positive subjects with determined HLA types but unavailable PBMCs (*P* = .03, by the log-rank test; HR, 0.29 [95% CI, .06–.99; *P* = .04, by a Cox regression model; Supplementary Table 2]). Finally, among the HLA-B*58:02–positive subjects studied, one individual also expressed protective HLA-B*58:01, and exclusion of this subject did not materially alter the findings described (viral set point difference, *P* = .01; time to a CD4^+^ T-cell count of <350 cells/mm^3^, *P* = .04). Similarly, no significant difference was observed with respect to HLA-C alleles expressed among the HLA-B*58:02–positive subjects studied; in each case, HLA-C*06:02 was expressed in linkage disequilibrium with HLA-B*58:02.

### HLA-Specific Vaccine Effects on Viral Load and CD4^+^ T-Cell Counts: Neither Protective nor Disease-Susceptible HLA Alleles

**Figure 3.**
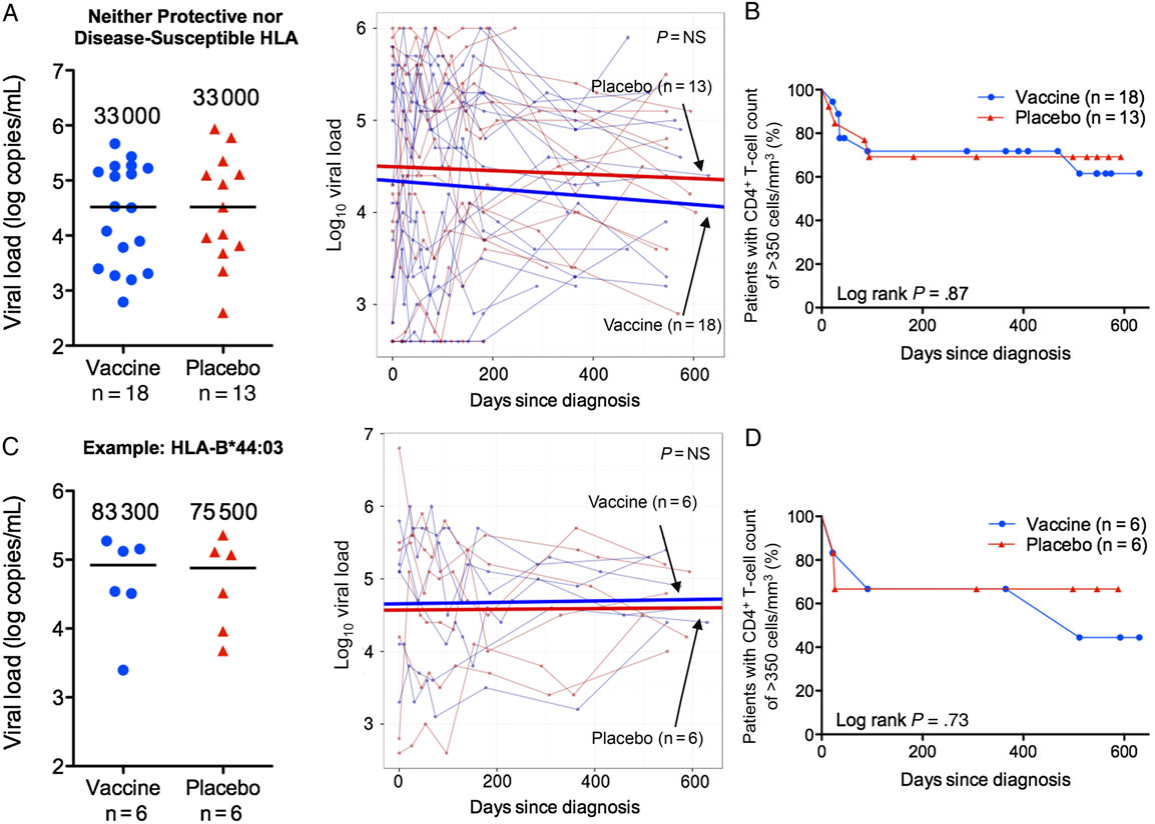
Chronic viral set point and CD4^+^ T-cell counts in the infected Phambili subjects expressing neither protective nor disease-susceptible HLA alleles. *A* and *B*, Data for the participants expressing neither protective nor disease-susceptible alleles overall (18 vaccinees and 13 placebo recipients). *C* and *D*, Example of a subset of the subjects from panels *A* and *B* who express HLA-B*44:03 and for whom there were sufficient subject numbers to compare vaccine (n = 6) and placebo (n = 6) groups. *A* and *C*, Chronic viral set point (defined for each individual as the median of all measurements from >3 months after diagnosis) is shown at left. Each patient's set point is shown as a circle (vaccine recipients) or triangle (placebo recipients), and horizontal lines and numbers denote median values for vaccine and placebo groups. Statistical analysis was performed by the Mann–Whitney *U* test. Examination of the effect of vaccination on longitudinal viral load values before antiretroviral therapy initiation is shown at right. The slopes (thick lines) generated from a linear mixed-effects model are plotted on top of the data points and viral load trajectories (thin lines) for individual patients. Statistical analysis was performed by analysis of variance. *B* and *D*, Kaplan–Meier curves showing the time to reach a CD4^+^ T-cell count of <350 cells/mm^3^ in vaccinees and placebo recipients. Statistical analysis was performed by the log-rank test. Abbreviation: NS, not significant.

Analysis of the subjects expressing neither protective nor disease-susceptible alleles showed no differences between vaccine and placebo groups in chronic viral set points or time to a CD4^+^ T-cell count of <350 cells/mm^3^ (Figure 3*A* and 3*B*). Representative observations are illustrated by the HLA-B*44:03–expressing subjects (6 in each group; Figure 3*C* and 3*D*).

### HLA-Specific Vaccine Effects on Anti–HIV-1 CD8^+^ T-Cell Responses

**Figure 4.**
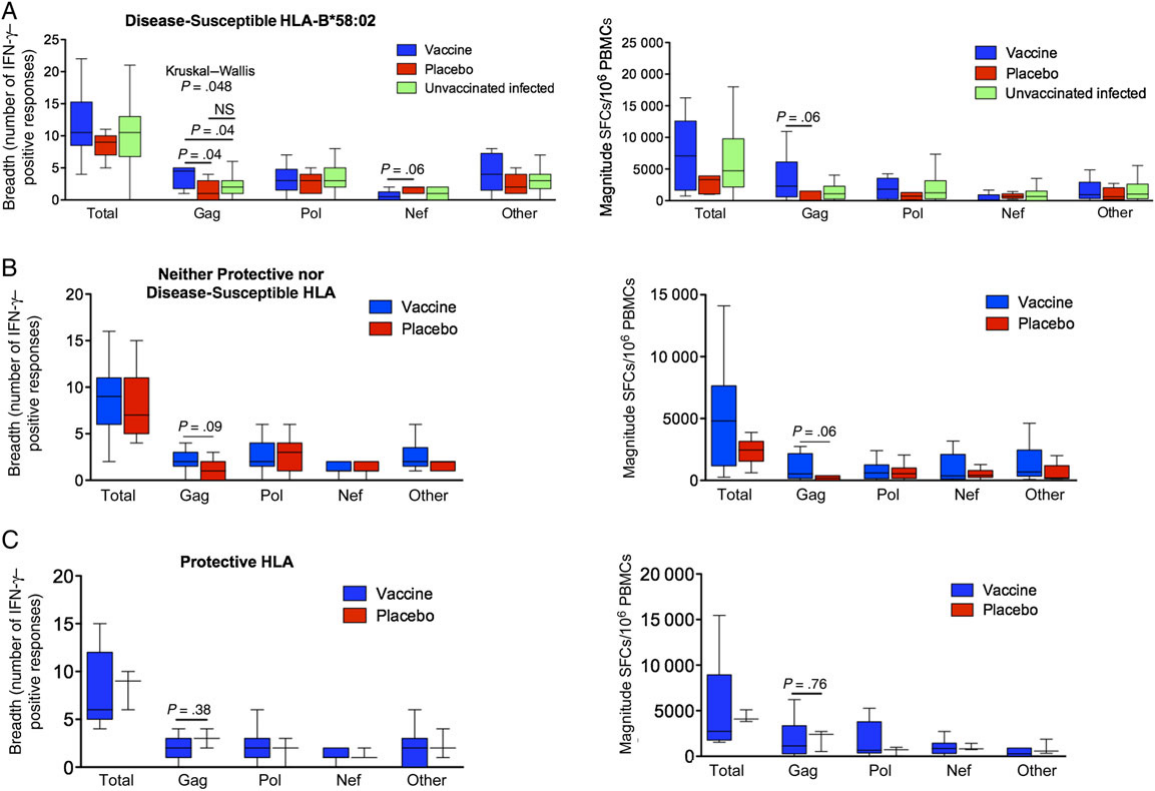
CD8^+^ T-cell responses in the infected Phambili subjects expressing disease-susceptible HLA-B*58:02 or those expressing neither protective nor disease-susceptible alleles or those expressing protective HLA alleles. *A*, Breadth and magnitude of CD8^+^ T-cell responses at the second time point in HLA-B*58:02–positive vaccinees, placebo recipients, and C clade chronically infected unvaccinated B*58:02-positive individuals. *B*, Breadth and magnitude of CD8^+^ T-cell responses at the second time point in vaccinees and placebo recipients expressing neither protective nor disease-susceptible HLA-B alleles. *C*, Breadth and magnitude of CD8^+^ T-cell responses at the second time point in vaccinees and placebo recipients expressing protective HLA-B alleles. In Tukey box plots, horizontal lines indicate median values, boxes show interquartile ranges, and whiskers show minimum and maximum values. Statistical analysis was performed by the Kruskal–Wallis and Mann–Whitney *U* tests. The column termed “Total” shows the combined values of responses to all proteins; the column termed “Other” includes responses to Env, Rev, Tat, Vpu, Vif, and Vpr. Abbreviations: IFN-γ, interferon γ; NS, not significant; PBMC, peripheral blood mononuclear cell; SFC, spot-forming cells.

To investigate whether the apparent effects on reduced viral load and slower time to a CD4^+^ T-cell count of <350 cells/mm^3^ observed might be related to HLA-specific induction of altered CD8^+^ T-cell responses in the vaccinees versus placebo recipients, we first analyzed these in HLA-B*58:02–positive study subjects. Median Gag-specific breadth was 4.5-fold higher in vaccinees (4.5 responses [IQR, 2–5 responses] vs 1 response [IQR, 0–3 responses]; *P* = .04; Figure 4*A*), and the median Gag-specific magnitude was 32-fold higher (2300 SFCs/10^6^ PBMCs [median, 603–6105 SFCs/10^6^ PBMCs] vs 70 SFCs/10^6^ PBMCs [0–1520 SFCs/10^6^ PBMCs]; *P* = .06; Figure 4*A*). Additionally, we compared the CD8^+^ T-cell responses in the Phambili subjects to those of HLA-B*58:02–positive unvaccinated individuals with natural chronic HIV-1 C-clade infection (n = 66; Figure 4*A*), most of whom (79%) were females recruited in South Africa (Supplementary Table 3). Here also we observed that the Phambili HLA-B*58:02–positive vaccinees had a significantly higher Gag-specific breadth as compared to non-Phambili infected subjects (*P* = .04), while the difference between HLA-B*58:02–positive placebo recipients and non-Phambili subjects was not significant. The median Gag-specific magnitude was >2-fold higher in HLA-B*58:02–positive vaccinees as compared to non-Phambili HLA-B*58:02–positive subjects (1100 SFCs/10^6^ PBMCs [IQR, 255–2050 SFCs/10^6^ PBMCs]; *P* = .2). No significant associations were found between Gag-specific breadth/magnitude and viral loads or CD4^+^ T-cell count in HLA-B*58:02–positive vaccinees (Supplementary Figure 2).

In contrast, in subjects expressing neither protective nor disease-susceptible alleles, the difference between the groups in median Gag-specific breadth and magnitude was more modest (2 responses [IQR, 1.5–3 responses] vs 1 response [IQR, 0–2 responses; *P* = .09]; and 529 SFCs/10^6^ PBMCs [IQR, 166–2163 SFCs/10^6^ PBMCs] vs 180 SFCs/10^6^ PBMCs [IQR, 0–377 SFCs/10^6^ PBMCs; *P* = .06]; Figure 4*B*). No significant differences were observed in Gag breadth and magnitude in the subjects expressing protective HLA (Figure 4*C*).

Although the MRKAd5 HIV-1 Gag/Pol/Nef vaccine appeared to boost Gag-specific responses overall in vaccinees within the 60 subjects studied here (Figure 1*D*), there were no significant differences for the CD8^+^ T-cell responses toward Pol, Nef, or Env, Vif, Vpr, Vpu, Tat, or Rev in the 3 HLA groups (Figure 4). However, of note, in addition to having a significantly broader Gag-specific response, HLA-B*58:02–positive vaccinees tended to have a narrower Nef-specific response (*P* = .06; Figure 4*A*). The potential relevance of this is discussed below.

**Figure 5.**
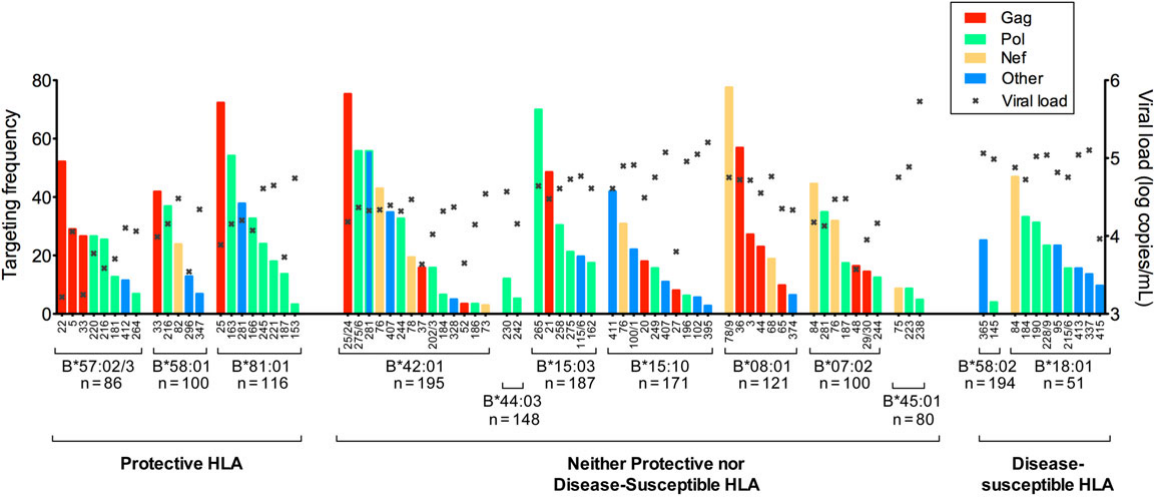
HLA-dependent differential immunodominance hierarchy of CD8^+^ T-cell responses in chronically C clade–infected, antiretroviral-naive, South African adults. CD8^+^ T-cell immunodominance hierarchy in subjects in the 3 HLA groups (allele frequency ≥5%), expressing protective HLA, neither protective nor disease-susceptible HLA, and disease-susceptible HLA. Targeting frequency denotes the percentage of subjects making detectable responses in interferon γ enzyme-linked immunospot assays to the named human immunodeficiency virus type 1 peptide. Numbers on the *x*-axis refer to the particular overlapping peptide (of 410 spanning the C clade proteome) to which a response was detected. The x in each column denotes the median viral load of responders to that peptide.

Together, our findings support the original hypothesis that the MRKAd5 HIV-1 vaccine could improve disease outcome by boosting beneficial Gag-specific responses [5] in HLA-B*58:02–positive individuals who, during naturally acquired infection, do not generate HLA-B*58:02–restricted Gag responses but produce predominantly Env-specific ineffective responses (Figure 5).

## DISCUSSION

This study set out to investigate whether the HLA-specific vaccine effects suggested in the Step trial [12, 13, 28] could be demonstrated in the ethnically dissimilar Phambili cohort. Half of the Step trial participants who subsequently became infected were white, whereas none of the Phambili cohort was white. Expression of HLA class I molecules, such as HLA-B*42:01, HLA-B*58:02, and HLA-B*81:01, for example, is observed in 0% of white individuals [29] and in 20%, 23%, and 10%, respectively, of Black South Africans [2, 5]. The principal finding in the current study is an HLA-specific vaccine effect in subjects expressing the disease-susceptible HLA-B*58:02. HLA-B*58:02–positive vaccinees had a significantly lower viral set point and progressed more slowly to a CD4^+^ T-cell count of <350 cells/mm^3^, compared with HLA-matched placebo recipients. This result was associated with an increased breadth and magnitude of Gag-specific CD8^+^ T-cell responses in vaccinees expressing HLA-B*58:02. In contrast, in Phambili subjects expressing neither protective nor disease-susceptible alleles, these vaccine effects were not observed.

The described HLA-specific vaccine effect was suggested in the Step study in relation to the protective HLA-B*27:05/B*57:01/B*58:01 [12, 13, 28]. Analysis here also showed a 1.3 log_10_ lower viral load and significantly slower progression to CD4^+^ T-cell count of <350 cells/mm^3^ (*P* = .009) in vaccinees expressing protective HLA-B*57:02/57:03/58:01/81:01 [2, 3], compared with HLA-matched placebo recipients. However, these analyses were limited by numbers (only 3 placebo recipients).

Our finding of reduced viral load and slower time to a CD4^+^ T-cell count of <350 cells/mm^3^ in HLA-B*58:02–positive vaccinees is significant. First, viral load in natural infection is 4.9–5.2 log_10_ copies/mL [6, 30], similar to that in the HLA-B*58:02–positive placebo recipients here (5.1 log_10_ copies/mL), corresponding to rapid progression and a high risk of further transmissions [2–4, 31]. The 17-fold reduction in viral set point in HLA-B*58:02–positive vaccinees observed here equates to a >5-year AIDS-free period without ART and a >2-fold reduction in onward transmission risk [31]. Second, HLA-B*58:02 is highly prevalent in sub-Saharan Africa (20%–25% of South Africans) [2, 5]. Thus, a vaccine effect on postinfection disease progression in subjects expressing protective HLA-B*57/58:01/81:01 (24% of South Africans [2, 5]) and in those expressing HLA-B*58:02 would favorably affect disease course in approximately half of the population.

The HLA-B*58:02–specific effect observed supports the original hypothesis. HLA-B*58:02–restricted CD8^+^ T-cell responses are almost exclusively directed against Env, which is associated with a high viral load [5] (Figure 5). The MRKAd5 HIV-1 vaccine might therefore be expected to improve outcome by boosting beneficial Gag responses [5, 11]. A 70-fold higher magnitude and a 4–5-fold greater breadth of Gag-specific responses were observed in HLA-B*58:02–positive vaccine recipients, but the necessary samples were unavailable to determine whether these were restricted by HLA-B*58:02 or by other HLA alleles expressed by these subjects.

Although previous studies have shown a correlation between increasing Gag breadth and decreasing viral load [5, 15, 32, 33], including in vaccinees in the Step study [28], this was not observed here (Supplementary Figures 2 and 3). In part this is due to our small study numbers. In addition, Gag-specific responses are not equally potent. For example, p24-specific responses are associated with better viremic control than p17- or p15-specific responses [34]. However, in this study the design of the Gag peptide pools made these subanalyses not possible. Furthermore, sample nonavailability prevented the HLA restriction of the CD8^+^ T-cell responses in the vaccinees to be determined. In large cohort studies, on average, Gag-specific breadth is associated with a lower viral load, but in small studies the variation between the different Gag and non-Gag responses will strongly influence these analyses. Furthermore, the vaccine-mediated increase in Gag breadth may be associated with changes in the non–Gag-specific responses. On average Nef-specific and Env-specific responses are associated with higher viral loads [5, 15]. In our study, Nef-specific breadth in infected HLA-B*58:02–positive vaccinees was 4-fold lower than in HLA-B*58:02–positive placebo recipients (*P* = .06). Nef-specific breath has been consistently shown in previous studies to be associated with higher viral loads and the same observation is made here (Supplementary Figures 2 and 3). Thus, the finding of a lower viral loads in B*58:02-positive subjects with both higher Gag-specific and lower Nef-specific responses may be the result of a combination of these effects.

The limitations of this study include small subject numbers, the potential for postrandomization bias [35], and the caveats that apply to any post hoc analysis. To address the possibility that the differences in postinfection outcomes were caused by the differences in characteristics associated with infection risk, rather than by vaccination, we investigated the impact of HLA on HIV-1 acquisition among 329 (of 801) Phambili subjects with available HLA data. There was no such influence in either vaccine or placebo recipients. This suggests that HLA does not confound the association of vaccination status with disease progression [36] and is consistent with ample data showing the impact of HLA on disease progression but not on HIV-1 acquisition [1, 37]. Skewing could have been introduced by unintended sample selection bias: PBMCs were only available for 60 of the 100 HIV-1–infected subjects, and the 40 subjects with unavailable samples and thus unknown CD8^+^ T-cell responses showed a faster time to a CD4^+^ T-cell count of <350 cells/mm^3^. However, the absence of data on the CD8^+^ T-cell response in this group of 40 more-rapidly progressing subjects does not alter the validity of the finding in relation to the 60 we were able to study.

Finally, although low CD4^+^ T-cell count and high viral load are, separately, strongly correlated with an increased risk of opportunistic infections and mortality [38, 39], these measures do not necessarily equate with disease [40]. However, the observation of progression to a CD4^+^ T-cell count of <350 cells/mm^3^ within 6 months of infection in >80% of the HLA-B*58:02–positive placebo recipients, together with a viral set point of 5.1 log_10_ copies/mL in these subjects, is consistent with rapid progression observed in natural infection in HLA-B*58:02–positive individuals [6, 30]. The 1.3 log_10_ lower viral set point in HLA-B*58:02–positive vaccinees and the inability of >50% to meet the CD4^+^ T-cell count criteria for ART initiation by 600 days after infection are consistent with vaccine-mediated protection against rapid progression.

In conclusion, although the MRKAd5 HIV-1 Gag/Pol/Nef vaccine increased the infection risk among vaccine recipients in the Step and Phambili trials [7, 8, 10], these data suggest its potential role as a therapeutic vaccine operating in an HLA-specific manner, with the capacity to benefit individuals expressing disease-susceptible alleles. Even in ART recipients, CD8^+^ T cells may contribute to suppression of viremia [41], and so-called shock and kill cure strategies [42] may likely require effective anti–HIV-1 CD8^+^ T-cell activity to achieve successful eradication of viral reservoirs [43]. Any theoretical increased risk of HIV-1 infection resulting from adenoviral-based vaccines in subjects already infected with HIV-1 would be low as compared to the potential therapeutic benefit of inducing an effective anti–HIV-1 T-cell response. These studies indicate that the dramatic differences in HLA composition between distinct populations may contribute to variation in vaccine efficacy.

## Supplementary Data

Supplementary materials are available at http://jid.oxfordjournals.org. Consisting of data provided by the author to benefit the reader, the posted materials are not copyedited and are the sole responsibility of the author, so questions or comments should be addressed to the author.

Supplementary Data
