# Supplementary material for: Lower Viral Loads and Slower CD4+ T-Cell Count Decline in MRKAd5 HIV-1 Vaccinees Expressing Disease-Susceptible HLA-B*58:02
Source: J Infect Dis. 2016 Mar 6;214(3):379–89. doi: 10.1093/infdis/jiw093 (PMC4936641; doi:10.1093/infdis/jiw093)
Supplement: Supplementary Data [file supp_214_3_379__index.html]

Lower Viral Loads and Slower CD4 Decline in MRKAd5 HIV-1 Vaccinees Expressing Disease-Susceptible HLA-B\*58:02 — Lower Viral Loads and Slower CD4+ T-Cell Count Decline in MRKAd5 HIV-1 Vaccinees Expressing Disease-Susceptible HLA-B\*58:02 — Lower Viral Loads and Slower CD4+ T-Cell Count Decline in MRKAd5 HIV-1 Vaccinees Expressing Disease-Susceptible HLA-B\*58:02 — Supplementary Data 

# Lower Viral Loads and Slower CD4+ T-Cell Count Decline in MRKAd5 HIV-1 Vaccinees Expressing Disease-Susceptible HLA-B\*58:02

## Supplementary Data

Supplementary Data

- Supplementary Data - Pdf file
