# Supplementary material for: Lower Viral Loads and Slower CD4+ T-Cell Count Decline in MRKAd5 HIV-1 Vaccinees Expressing Disease-Susceptible HLA-B*58:02
Source: J Infect Dis. 2016 Mar 6;214(3):379–89. doi: 10.1093/infdis/jiw093 (PMC4936641; doi:10.1093/infdis/jiw093)
Supplement: Supplementary Data [file supp_jiw093_jiw093supp.pdf]

SUPPLEMENTARY DATA

LOWER VIRAL LOADS AND SLOWER CD4 DECLINE IN MRKAD5 HIV-1 VACCINEES EXPRESSING DISEASE-SUSCEPTIBLE HLA-B*58:02

Ellen M. Leitman, Jacob Hurst, Masahiko Mori, James Kublin, Thumbi Ndung'u, Bruce D. Walker, Jonathan Carlson, Glenda E. Gray, Philippa C. Matthews, Nicole Frahm, Philip J. R. Goulder

EXTENDED METHODS

Study design

Our Phambili Ancillary study investigated HLA-specific effects on disease progression and CD8+ T-cell immune responses in the Phambili trial participants who subsequently became HIV-1-infected. Phambili, described previously [1, 2], was a double-blind placebo-controlled randomized phase 2b trial that enrolled 801 heterosexual HIV-1-negative South Africans aged 18-35 years, from January to September 2007 (ClinicalTrials.gov: NCT00413725, South African Research Database: DOH-27-0207-1539). There were 100 HIV-1 infections diagnosed after enrollment in the Phambili cohort by 42 months follow-up [2]. These subjects were divided into two groups for analysis: (i) 60 subjects, for whom HLA types, PBMC, viral load, and CD4 data were available; and (ii) an additional 40 subjects for whom viral load and CD4 data but no PBMC were available. In this second group, material for HLA typing was available for only 25 subjects (Supplementary Figure 1).

HLA typing was determined for 85 subjects from whom material was available using a locus specific PCR amplification strategy and a heterozygous DNA sequencing methodology for exon 2 and 3 of the class I PCR amplicon [3]. Having categorized the individual by their HLA-B expression into those with ‘Protective’, ‘Disease-susceptible’ and ‘Neither Protective nor Disease-susceptible’, we compared viral loads, CD4+ T-cell counts and HIV-1-specific CD8+ T-cell responses (blinded to the vaccine/placebo treatment assignment) between vaccinees and placebo-recipients within each HLA group.

HIV-1 plasma viral load was determined using the Roche COBAS Amplicor Monitor HIV-1 Standard Ver 1.5, with COBAS AmpliPrep, with limits of detection below 400 and above 1,000,000 copies/ml.

Interferon-γ ELISPOT assays

ELISPOT assays were performed as previously described [4, 5]. Spots were counted using an automated ELISPOT reader (AID ELISPOT v4.0, Autoimmun Diagnostika, Germany). Breadth was defined as the number of pools testing positive (>50 SFC/10^6PBMC after background subtraction). All assays were carried out blinded to the vaccine/placebo treatment assignments. For the 60 Phambili subjects, for whom cells were available, PBMC from 59 were screened because cells from one placebo-recipient were not viable upon thawing. Additionally, CD8+ T-cell responses of five subjects at the second timepoint were excluded from the analyses because ART had been initiated.

Statistical analysis

We used a decision tree based on Fisher's Exact Test to identify associations between expression of HLA alleles and recognition of HIV-1-specific peptides, as previously described [6]. For each peptide, we computed the Fisher's Exact Test p-value against all four-digit HLA alleles observed in the Sinikithemba and Gateway cohorts and added the most significant HLA allele to the decision tree. We next removed all individuals who expressed that allele and repeated the process until the most significant HLA allele had a p-value of >0.2. False-discovery rates [7] were calculated using a procedure-specific

to Fisher's Exact Test that analytically computes the null distribution for all permutation of the data, as previously described [8].

Spearman correlation analyses of breadth or magnitude of CD8+ T-cell responses with either viral loads or CD4 counts were performed in Prism (v5.0c, GraphPad).

SUPPLEMENTARY FIGURE LEGENDS AND TABLES

Supplementary Figure 1. Phambili ancillary study profile. [a] These 60 subjects are presented in Tables 1 and 2. [b] Material available for HLA typing on 25 subjects. [c] One placebo-recipient, co-expressing protective HLA-B*58:01 and disease-susceptible HLA-B*58:02, is included in both HLA subsets in this figure.

Supplementary Figure 2. Correlations of Gag- and Nef-specific breadth and magnitude with viral load or CD4 counts in all subjects irrespective of HLA expression and subjects expressing disease-susceptible HLA. In all panels, correlations with viral load are presented in the top rows and with CD4 counts in the bottom rows. **(A)** Correlations between Gag-specific breadth and viral load or CD4 counts in vaccinees and placebo-recipients. **(B)** Correlations between Nef-specific breadth and viral load or CD4 counts in vaccinees and placebo-recipients. **(C)** Correlations between Gag-specific magnitude and viral load or CD4 counts in vaccinees and placebo-recipients. **(D)** Correlations between Nef-specific magnitude and viral load or CD4 counts in vaccinees and placebo-recipients. P and r-values were obtained from Spearman correlation analyses.

Supplementary Figure 3. Correlations of Gag- and Nef-specific breadth and magnitude with viral load or CD4 counts in subjects expressing protective HLA and neither protective nor disease-susceptible HLA. In all panels, correlations with viral load are presented in the top rows and with CD4 counts in the bottom rows. **(A)** Correlations between Gag-specific breadth and viral load or CD4 counts in vaccinees and placebo-recipients. **(B)** Correlations between Nef-specific breadth and viral load or CD4 counts in vaccinees and placebo-recipients. **(C)** Correlations between Gag-specific magnitude and viral load or CD4 counts in vaccinees and placebo-recipients. **(D)** Correlations between Nef-specific magnitude and viral load or CD4 counts in vaccinees and placebo-recipients. P and r-values were obtained from Spearman correlation analyses.

Supplementary Table 1. Univariate Cox regression model comparing time to reach CD4+ T-cell count of <350 cells/mm^3 in vaccinees versus placebo-recipients expressing protective HLA-B*57/58:01/81:01. Ad5 denotes adenovirus type 5; HSV-2 denotes herpes simplex virus 2; HR denotes hazard ratio; CI denotes confidence interval.

Supplementary Table 2. Univariate Cox regression model comparing time to reach CD4+ T-cell count of <350 cells/mm^3 in vaccinees versus placebo-recipients expressing disease-susceptible HLA-B*58:02. Ad5 denotes adenovirus type 5; HSV-

2 denotes herpes simplex virus 2; HR denotes hazard ratio; CI denotes confidence interval.

Supplementary Table 3. Characteristics of the unvaccinated ART-naïve HLA-B*58:02-positive C-clade infected subjects outside of the trial (n=66). [a] CD8+ T-cell ELISPOT assays, viral load and CD4+ T-cell measurements were performed at enrollment. IQR denotes interquartile range.

SUPPLEMENTARY REFERENCES

1. Gray GE, Allen M, Moodie Z, et al. Safety and efficacy of the HVTN 503/Phambili study of a clade-B-based HIV-1 vaccine in South Africa: a double-blind, randomised, placebo-controlled test-of-concept phase 2b study. Lancet Infect Dis **2011**; 11:507-15.

2. Gray GE, Moodie Z, Metch B, et al. Recombinant adenovirus type 5 HIV gag/pol/nef vaccine in South Africa: unblinded, long-term follow-up of the phase 2b HVTN 503/Phambili study. The Lancet Infectious diseases **2014**; 14:388-96.

3. Cano P, Klitz W, Mack SJ, et al. Common and well-documented HLA alleles: report of the Ad-Hoc committee of the american society for histocompatiblity and immunogenetics. Human immunology **2007**; 68:392-417.

4. Altfeld MA, Trocha A, Eldridge RL, et al. Identification of dominant optimal HLA-B60- and HLA-B61-restricted cytotoxic T-lymphocyte (CTL) epitopes: rapid characterization of CTL responses by enzyme-linked immunospot assay. J Virol **2000**; 74:8541-9.

5. Addo MM, Yu XG, Rathod A, et al. Comprehensive epitope analysis of human immunodeficiency virus type 1 (HIV-1)-specific T-cell responses directed against the entire expressed HIV-1 genome demonstrate broadly directed responses, but no correlation to viral load. J Virol **2003**; 77:2081-92.

6. Kloverpris HN, Harndahl M, Leslie AJ, et al. HIV control through a single nucleotide on the HLA-B locus. J Virol **2012**; 86:11493-500.

7. Benjamini Y, Hochberg, Y. Controlling the false discovery rate: a practical and powerful approach to multiple testing. Journal of the Royal Statistical Society Series B **1995**; 57:289-300.

8. Carlson J, Heckerman, D., Shani, G. Estimating false discovery rates for contingency tables. Microsoft, Redmond, WA **2009**.

Supplementary Figure 1

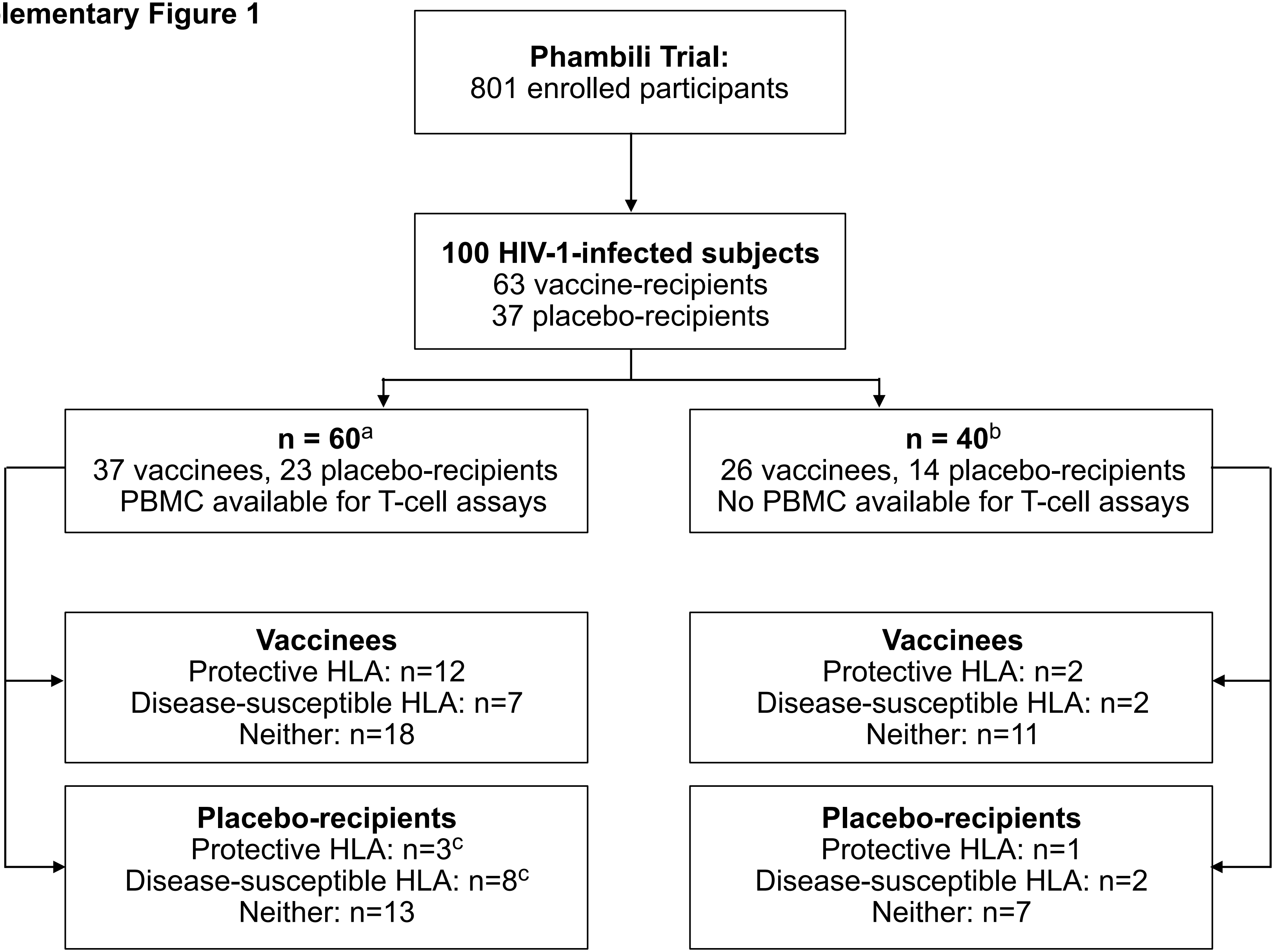

[a] These 60 subjects are presented in Tables 1 and 2.

[b] Material available for HLA typing on 25 subjects.

[c] One placebo-recipient, co-expressing protective HLA-B*58:01 and disease-susceptible HLA-B*58:02, is included in both HLA subsets in this figure.

Supplementary Figure 2

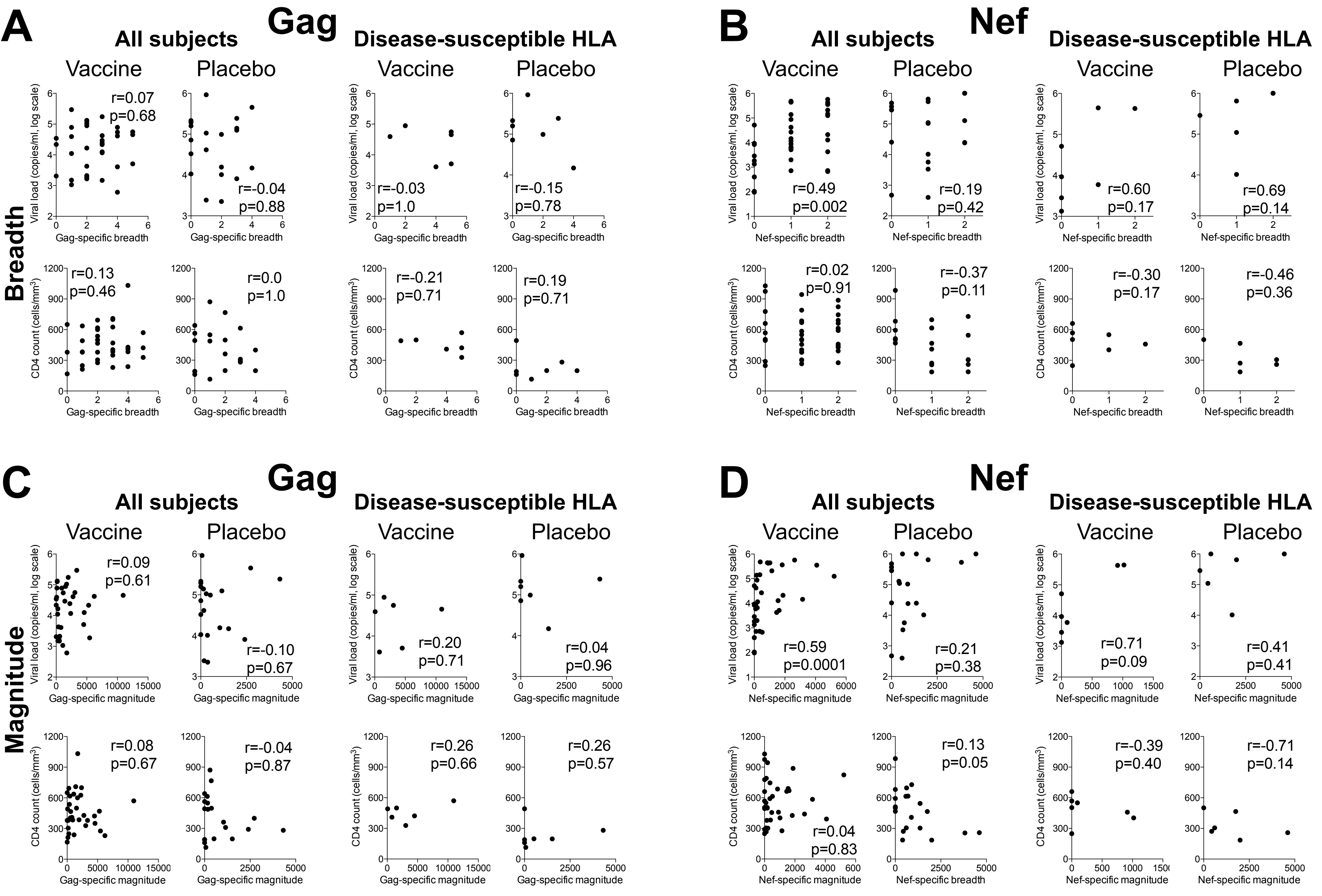

Supplementary Figure 3

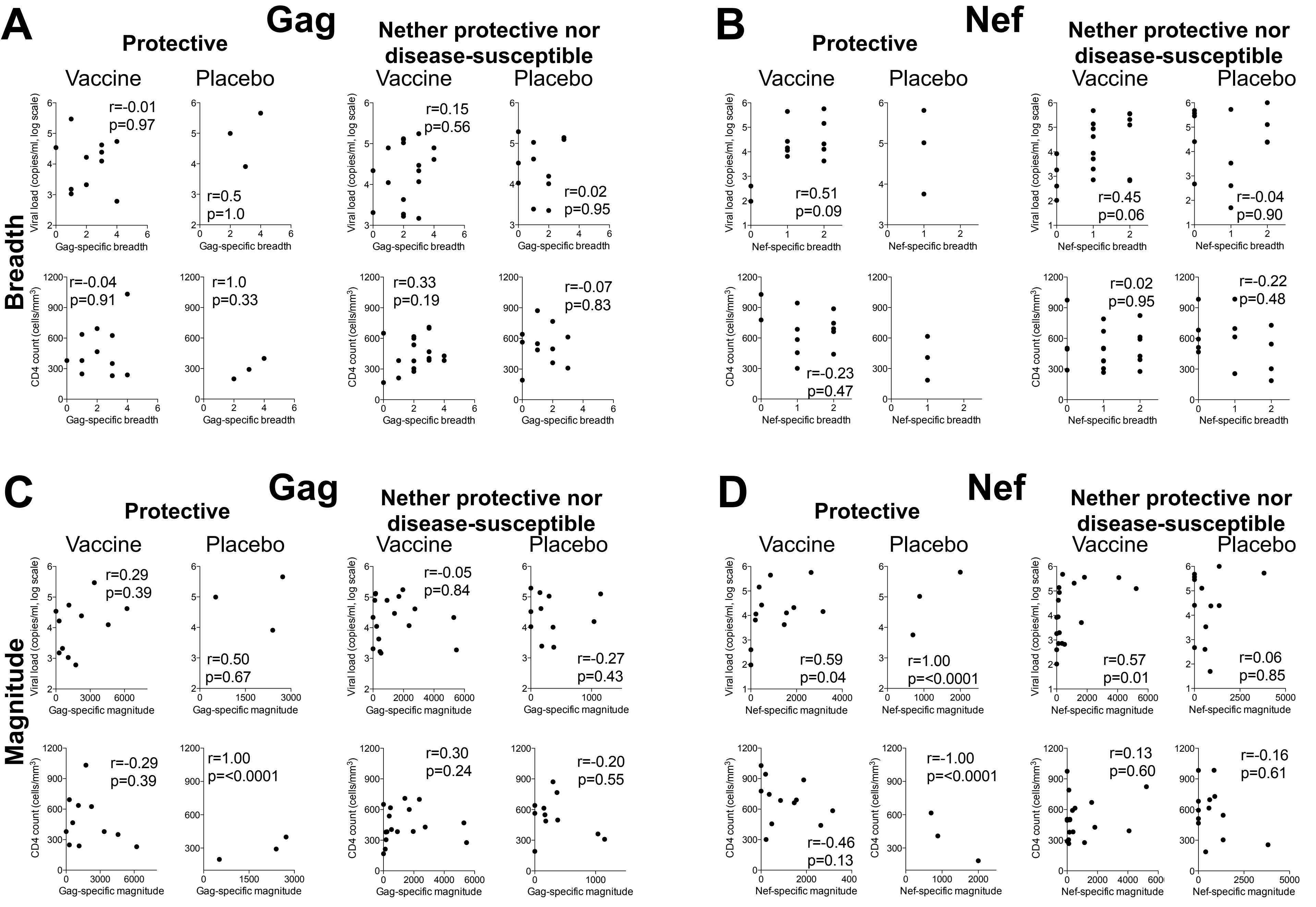

Supplementary Table 1. Univariate Cox regression model comparing time to reach CD4+ T-cell count of <350 cells/mm^3 in vaccinees versus placebo-recipients expressing protective HLA-B*57/58:01/81:01.

Variable (indicator)	HR (95% CI)	p value
**Protective HLA: vaccinees (n=12) and placebo-recipients (n=3) out of 60 (univariate Cox)**		
Placebo- or vaccine-treatment arm (vaccine)	0.17 (0.04-0.78)	0.02
Sex (female)	1.27 (0.31-5.17)	0.74
Age (continuous)	1.12 (0.96-1.30)	0.17
Ad5 (Ad5>18)	2.59 (0.32-20.41)	0.38
HSV-2 at enrollment (HSV-2+)	0.45 (0.11-1.86)	0.27

Ad5 denotes adenovirus type 5; HSV-2 denotes herpes simplex virus 2; HR denotes hazard ratio; CI denotes confidence interval.

Supplementary Table 2. Univariate Cox regression model comparing time to reach CD4+ T-cell count of <350 cells/mm^3 in vaccinees versus placebo-recipients expressing disease-susceptible HLA-B*58:02.

Variable (indicator)	HR (95% CI)	p value
**HLA-B*58:02-positive vaccinees (n=7) and placebo-recipients (n=7) out of 60 (univariate Cox)**		
Placebo- or vaccine-treatment arm (vaccine)	0.22 (0.05-0.91)	0.04
Sex (female)	1.90 (0.39-9.26)	0.43
Age (continuous)	1.05 (0.93-1.18)	0.44
Ad5 (Ad5>18)	0.56 (0.14-2.32)	0.44
HSV-2 at enrollment (HSV2+)	1.69 (0.45-6.39)	0.44
**HLA-B*58:02-positive vaccinees (n=8) and placebo-recipients (n=9) out of 100 (univariate Cox)**		
Placebo- or vaccine-treatment arm (vaccine)	0.29 (0.06-0.99)	0.04
Sex (female)	1.55 (0.33-7.34)	0.58
Age (continuous)	1.01 (0.89-1.15)	0.84
Ad5 (Ad5>18)	0.52 (0.13-2.04)	0.35
HSV-2 at enrollment (HSV-2+)	1.28 (0.36-4.56)	0.70

Ad5 denotes adenovirus type 5; HSV-2 denotes herpes simplex virus 2; HR denotes hazard ratio; CI denotes confidence interval.

Supplementary Table 3. Characteristics of the unvaccinated ART-naïve HLA-B*58:02-positive C-clade infected subjects outside of the trial (n=66).

Characteristic	N (%)
Sex	52 (79%)
Female	10 (15%)
Male	4 (6%)
Unknown	
Country of origin	54 (82%)
South Africa	8 (12%)
Zimbabwe	2 (3%)
Somalia	1 (1.5%)
Malawi	1 (1.5%)
Kenya	
Year of enrollment[a]	19 (29%)
2003	18 (27%)
2004	14 (21%)
2005	3 (4.5%)
2006	6 (9%)
2007	6 (9%)
2008	
Age at enrollment (years), median (IQR)	31 (27-35)
Viral load (copies/ml), median (IQR)	52,850 (9,678-229,250)
CD4 count (cells/mm^3), median (IQR)	337 (227-431)

[a] CD8+ T-cell ELISPOT assays, viral load and CD4+ T-cell measurements were performed at enrollment. IQR denotes interquartile range.
